# Does Reactance against Cigarette Warning Labels Matter? Warning Label Responses and Downstream Smoking Cessation amongst Adult Smokers in Australia, Canada, Mexico and the United States

**DOI:** 10.1371/journal.pone.0159245

**Published:** 2016-07-13

**Authors:** Yoo Jin Cho, James F. Thrasher, Kamala Swayampakala, Hua-Hie Yong, Robert McKeever, David Hammond, Dien Anshari, K. Michael Cummings, Ron Borland

**Affiliations:** 1 Department of Health Promotion, Education, and Behavior, University of South Carolina, Columbia, South Carolina, United States of America; 2 Department of Tobacco Research, Center for Population Health Research, National Institute of Public Health, Cuernavaca, Mexico; 3 Cancer Council Victoria, Melbourne, Victoria, Australia; 4 School of Journalism and Mass Communications, University of South Carolina, Columbia, South Carolina, United States of America; 5 School of Public Health & Health Systems, University of Waterloo, Waterloo, Ontario, Canada; 6 Department of Health Education & Behavioral Sciences, Faculty of Public Health, Universitas Indonesia, Depok, Indonesia; 7 Department of Psychiatry & Behavioral Sciences, Medical University of South Carolina, Charleston, South Carolina, United States of America; Geisel School of Medicine at Dartmouth College, UNITED STATES

## Abstract

**Objective:**

Some researchers have raised concerns that pictorial health warning labels (HWLs) on cigarette packages may lead to message rejection and reduced effectiveness of HWL messages. This study aimed to determine how state reactance (i.e., negative affect due to perceived manipulation) in response to both pictorial and text-only HWLs is associated with other types of HWL responses and with subsequent cessation attempts.

**Methods:**

Survey data were collected every 4 months between September 2013 and 2014 from online panels of adult smokers in Australia, Canada, Mexico, and the US were analyzed. Participants with at least one wave of follow-up were included in the analysis (n = 4,072 smokers; 7,459 observations). Surveys assessed psychological and behavioral responses to HWLs (i.e., attention to HWLs, cognitive elaboration of risks due to HWLs, avoiding HWLs, and forgoing cigarettes because of HWLs) and cessation attempts. Participants then viewed specific HWLs from their countries and were queried about affective state reactance. Logistic and linear Generalized Estimating Equation (GEE) models regressed each of the psychological and behavioral HWL responses on reactance, while controlling for socio-demographic and smoking-related variables. Logistic GEE models also regressed having attempted to quit by the subsequent survey on reactance, each of the psychological and behavioral HWL responses (analyzed separately), adjustment variables. Data from all countries were initially pooled, with interactions between country and reactance assessed; when interactions were statistically significant, country-stratified models were estimated.

**Results:**

Interactions between country and reactance were found in all models that regressed psychological and behavioral HWL responses on study variables. In the US, stronger reactance was associated with more frequent reading of HWLs and thinking about health risks. Smokers from all four countries with stronger reactance reported greater likelihood of avoiding warnings and forgoing cigarettes due to warnings, although the association appeared stronger in the US. Both stronger HWLs responses and reactance were positively associated with subsequent cessation attempts, with no significant interaction between country and reactance.

**Conclusions:**

Reactance towards HWLs does not appear to interfere with quitting, which is consistent with its being an indicator of concern, not a systematic effort to avoid HWL message engagement.

## Introduction

Article 11 of the World Health Organization Framework Convention on Tobacco Control recommends that parties implement pictorial health warning labels (HWLs) to increase public awareness about tobacco risks [1]. In addition to increasing awareness, pictorial health warnings can significantly reduce public tobacco consumption by increasing smokers’ intentions to quit, quit attempts, and quitting [2–5]. Compared to text warnings, pictorial HWLs appear more effective in increasing attention to HWLs, cognitive elaboration, and intentions to quit [6–8], with some evidence to support enhanced impact among smokers from lower socioeconomic groups [9, 10].

Some researchers have criticized pictorial HWLs based on fear appeal theories, arguing that smokers may not attend to HWLs with fear-arousing messages and even when they attend to HWLs, they may react defensively towards them [11]. The extended parallel process model (EPPM) presents a theoretical framework for fear appeal research [12], characterizing fear appeals as persuasive messages that present a threat to arouse fear and then provide recommendations to deter the threat. According to the EPPM, fear appeals lead people with low perceived efficacy (e.g., beliefs about the benefits of, or ability to follow, the recommended action) to have message rejection responses, such as reactance. For instance, smokers who believe that they do not have the ability to quit smoking may minimize HWLs (i.e. think that HWLs are “distorted” or “overstated”) to control their fear [13].

Psychological reactance theory predicts that people will reject persuasive messages when they feel their freedom is threatened [14, 15]. A primary assumption of reactance theory is that persuasive messages that aim to change behavior conflict with desires for autonomy, individuality, or self-determination [16, 17]. Some people appear to have stronger propensity to experience reactance when presented with persuasive messages, leading some researchers to examine reactance as a trait [16–20]. Smokers with the reactance trait should be particularly likely to experience reactance against HWLs that they perceive as aiming to persuade them not to smoke. State reactance, the situation-specific experience of perceived efforts to limit behavioral freedom, appears best explained by the Intertwined Process Model (IPM) [21]. The IPM posits that state reactance is comprised of both an affective and a cognitive dimension that mediate message effects on message rejection and behavioral intentions. Affective state reactance primarily concerns anger in response to perceived manipulation or pressure to do something the person is not prepared to do at the time, which appears to be the primary mechanism through which reactance exerts its effects [22, 23].

Experimental studies have produced somewhat inconsistent results regarding whether pictorial HWLs that graphically illustrate smoking-related diseases elicit responses that are argued to be maladaptive and whether such responses negatively influence intentions to quit smoking. For instance, one eye tracking study found that daily smokers tended to avoid pictorial HWLs on packs by fixating on branded or blank areas of the cigarette pack [24]. The cross-sectional study, however, could not examine downstream effects of avoidance. Moreover, according to Peters et al., while pictorial HWLs elicited more negative affect than text-only HWLs, pictorial HWLs were as likely as text-only HWLs to be attended, deemed credible, and supported by smokers [25]. Indeed, some research suggests that negative affect (i.e., worry and concern) that smokers report when viewing pictorial HWLs mediates HWL effects on risk perceptions [26] and promotes intentions to quit [4].

The experimental evidence is limited by low ecological validity, as the studies described above focus on short-term exposures and impacts, rather than on cessation-related outcomes under natural conditions of long-term, repeated exposures to HWLs. Data from population-based, observational studies suggest that indications of potentially maladaptive responses to HWLs do not inhibit smoking cessation [27, 28]. Indeed, these responses may reflect an underlying concern about smoking, and quitting smoking may serve as the best strategy to resolve the dissonance between the HWL reminder of smoking-related risks and urge for freedom of choice [29]. For instance, while smokers’ reports of avoiding HWLs increased after implementing pictorial HWLs, smokers who avoided HWLs were equally likely to quit smoking as their counterparts, and stronger fear predicted cessation behavior at follow-up [28, 30].

The limited research on the effects of reactance towards HWLs in cessation behavior is far from definitive. For instance, experimental studies found that pictorial HWLs elicit more freedom threat perceptions and state reactance among smokers than text only HWLs [23, 31], without explaining whether reactance matters in terms of the effectiveness of pictorial HWLs. While an experimental study found that trait reactance does not moderate the effects of pictorial HWLs in decreasing intention to smoke or in increasing quit intentions among young adult smokers [32], longitudinal observational studies are necessary to examine the long-term effect of reactance on cessation behavior. To our knowledge, only one prior observational study has examined the effect of trait reactance in HWL responses, finding that trait reactance was only weakly and inconsistently associated with HWL responses, and it did not hamper subsequent quitting behavior [33]. However, trait reactance represents the general tendency to experience state reactance, not the state itself [21], and so may underestimate reactance effects in response to specific persuasive messages. Because trait and state reactance may only be weakly correlated [23], a focus on state reactance is required to better understand how reactance influences smokers’ responses to HWLs. Our study seeks to advance knowledge about reactance to HWLs by examining how affective state reactance towards HWLs is associated with HWL responses and subsequent quitting behavior, using a longitudinal, observational design.

Based on the EPPM and psychological reactance theory, we posit the following hypotheses:

H1: Smokers with relatively stronger affective state reactance will: (a) report weaker attention to HWLs and cognitive elaboration of risks due to HWLs; (b) be more likely to avoid HWLs; and (c) be less likely to forgo cigarettes due to HWLs.

H2: Smokers with relatively stronger affective state reactance will be less likely to make quit attempts at follow-up than those with weaker reactance.

H3: The effects of HWL responses on downstream cessation will differ by smokers’ level of affective state reactance.

In addition to testing the primary hypotheses above, the design of the current study involving four countries, three of which have innovative pictorial HWLs policies (Australia, Canada, Mexico), and one of which has had a series of text-only HWLs since 1966 (the United States), affords an opportunity to test some questions that could be drawn from previous literature. First, as described in the reviewed literature, some experimental evidence suggests pictorial HWLs elicit greater affective state reactance [23, 32], thus it is plausible that greater affective state reactance may be found among smokers in countries with pictorial HWLs relative to smokers in the U.S., where text-only HWLs are the legal status quo. Likewise, the nature of affective state reactance aroused by text-only HWLs may differ from that of affective state reactance aroused by pictorial HWLs, making a different effect on HWL responses. Therefore, we hypothesized the following:

H4: The relationship between affective state reactance and study outcomes (HWL responses, cessation) will be stronger for smokers in countries with novel pictorial HWLs (i.e., Australia, Canada, Mexico) than for smokers in countries with older, text-only HWLs (i.e., U.S.).

Also, some may question whether the effect of state reactance on cessation behavior differs by one’s level of self-efficacy based on the EPPM theory [13], although a recent study on trait reactance did not find such differences [33]. Hence, the study also sought to examine the following hypothesis:

H5: The effect of affective state reactance on subsequent quit attempts will differ by smokers’ level of self-efficacy.

## Methods

### Sample

Adult smokers in Australia, Canada, Mexico and the United States were recruited from online consumer panels from Global Market Insights [34]. Eligible participants were those who, at the time of study enrollment, were 18 to 64 years old, had smoked 100 or more cigarettes in their lifetime, and had smoked at least once in the previous month. Approximately 1000 participants were surveyed in each country every four months from September 2012 to September 2014. In the U.S., an oversample of 400 Latinos was also surveyed at each wave. The analytic sample was limited to four surveys between September 2013 and September 2014, when affective state reactance was queried. The sample was replenished with new eligible participants at each wave to address attrition and maintain sample size. Because of hypotheses regarding subsequent cessation attempts, eligibility was further limited to those who participated in at least two consecutive surveys, yielding a final analytic sample of 4,072 adult smokers (Australia = 963; Canada = 948; Mexico = 975; US = 1186) who provided 7,459 observations (Australia = 1889; Canada = 1787; Mexico = 1695; US = 2088). Written informed consent was obtained from all participants. All procedures were approved by the IRB at the University of South Carolina.

### Measures

#### Reactance

Participants in Australia, Canada and Mexico were shown eight HWLs that were on cigarette packs at the time of the survey, whereas US participants were shown all four HWLs on packs in the United States. After each HWL was presented in random order and rated (e.g., fear, motivation to quit), affective state reactance was measured with three items (i.e., “I feel angry while viewing health warnings on cigarette packs”; “I feel annoyed while viewing health warnings on cigarette packs”; “I feel irritated while viewing health warnings on cigarette packs.”) adapted from prior research [35]. A 7-point response scale was used, with “strongly disagree” and “strongly agree” at scale endpoints, and responses to all three items were averaged to create the affective state reactance scale. A fourth item from the original scale (i.e., “I feel aggravated while viewing health warnings on cigarette packs”) was eliminated after the first survey because it did not meaningfully contribute to reliability (i.e., range for four- vs. three-item scale: α = 0.92 to 0.93 vs. α = 0.90 to 0.91, across countries). The cognitive state reactance dimension was not assessed because of the difficulty of using standard measurement approaches, which would require collecting and coding open-responses in a reliable way for thousands of observations in an online survey administered in multiple languages.

While prior reactance studies have conducted manipulation checks by assessing perceived threat to freedom [21, 22], the use of perceptual checks to confirm the success of manipulated message content may neither be necessary nor methodologically appropriate [35]. Nonetheless, because perceived threat represents a theoretically relevant psychological state situated between HWL content exposure and reactance, an effort was also made to ascertain participants' perceived threat to freedom. This was accomplished by asking participants to rate their agreement with three statements adapted from Quick [36] (“Health warnings on cigarette packages try to make a decision for me”; “Health warnings on cigarette packages threaten my freedom to choose”; “Health warnings on cigarette packages try to manipulate me.”), using the same 7-point response format as the reactance scale. Reliability was good across countries (α = 0.80–0.84 across countries), and responses were averaged.

#### Psychological and behavioral responses to HWLs

We assessed two psychological responses to HWLs, attention to HWLs and cognitive elaboration of risks due to HWLs. Attention to HWLs was assessed by asking participants how often they had noticed and read HWLs in the prior month. People who indicated that they had noticed HWLs were asked: “In the last month, how often, if at all, have you read or looked closely at the warning labels on cigarette packages?” on the five-point Likert-type scale ranged from 1 (“never”) to 5 (“very often”). People who reported not noticing HWLs were coded as never attended to HWLs. Cognitive elaboration of risks due to HWLs was assessed by asking “To what extent do the warning labels make you think about the health risks of smoking?” with a 9-point Likert scale ranged from 1 (“not at all”) to 9 (“extremely”). Adapted from previous research [37], the responses were then categorized into tertiles as low, moderate, and high.

Two behavioral responses to HWLs, avoiding HWLs and forgoing cigarettes due to HWLs, were measured in the study. Avoiding HWLs was measured by asking participants if they had made any effort to avoid looking at or thinking about the warning labels, such as covering them up, keeping them out of sight, using a cigarette case, avoiding certain warnings, or any other means in the last month. Forgoing of cigarettes due to HWLs was measured by asking participants if, in the prior month, the HWLs had stopped them from having a cigarette when they were about to smoke, with a 4-point Likert scale ranged from 1 (“never”) to 4 (“many times”). As in previous research [27], responses to both behavioral reaction questions were dichotomized to indicate any compared to no behavior.

#### Quit attempts

Participants were classified as having made a quit attempt if they answered affirmatively to the question asking whether they had made any attempts to stop smoking within the prior four months, anchoring the question with the date from four months prior. Participants who reported that they had quit smoking at follow-up were also classified as having made a quit attempt.

#### Adjustment variables

To address potential confounders that may affect the relationship between affective state reactance and responses to HWLs, we assessed the following socio-demographic variables: age (18–24; 25–34; 35–44; 45–54; 55–64), gender, education (high school or less; some college or university; and university or more), and annual household income (Australia, Canada, United States: $29,999 or less; $30,000-$59,999; and $60,000 or more). For greater comparability, a different cutpoint was used to assess monthly household income in Mexico ($5,000 or less; $5,001 to $10,000; and $10,001 or more). To avoid model misspecification, we also assessed smoking-related characteristics, which were previously associated with HWL responses [33]. The Heaviness of Smoking Index (HSI) was used to assess participants’ nicotine dependence [38] by combining information on the number of cigarettes smoked per day and timing of the first cigarette of the day. Intentions to quit was measured by asking if participants were planning to quit smoking (within the next month; within the next 6 months; sometime in the future, beyond 6 months; not planning to quit; don’t know), with responses dichotomized into those with intentions to quit smoking within the next month or six months versus other responses. Self-efficacy to quit smoking was assessed with a standard question [39] “If you decided to give up smoking completely in the next 6 months, how sure are you that you would succeed?,” using a 9-point scale ranging from 1 (not at all sure) to 9 (extremely sure). The number of prior surveys completed by participants was also assessed to adjust for potential differences among samples.

### Analysis

Stata, v 13, was used for all analyses. Differences between U.S. samples and samples from other countries were assessed using chi-square and t-tests. Within country, t-tests were used to assess differences in affective state reactance for the analytic sample as compared to the sample that was excluded due to having only one survey wave. Bivariate and adjusted linear General Estimating Equations (GEE) models regressed attention to HWLs and cognitive elaboration of risks (thinking about risks) on reactance, survey wave, and adjustment variables. Similarly, logistic GEE models were estimated, regressing behavioral reactions to HWLs (avoidance, forgoing cigarettes due to HWLs) on reactance, survey wave, and adjustment variables. Initial analyses involved pooling data across all countries, with the final adjusted model including an interaction term between reactance and country indicators (with U.S. as reference). These interactions were statistically significant in all models, so analyses were rerun after stratifying the data by country.

Data were pooled across countries, and bivariate and adjusted logistic GEE models estimated correlates of making a quit attempt during the subsequent four months of follow-up. To better understand how quitting was associated with reactance in the presence of each of the four psychological and behavioral HWL responses, four separate adjusted models were estimated. All adjusted models included affective state reactance, country, survey wave, and the adjustment variables, but only one of four psychological and behavioral responses to HWL variables was included in each model. For each of these adjusted models, interactions between country (U.S. as reference) and affective state reactance were assessed; however, the interactions were not statistically significant in any model, so country-stratified models were not estimated. Finally, additional interactions were assessed, one at a time, between affective state reactance and self-efficacy and each of the four HWLs response variables.

To help assess whether biases due to loss to follow-up were likely to influence results, sensitivity analyses were conducted by calculating propensity scores based on the probabilities of participating in only one wave versus two, three, or all four waves. Propensity scores were calculated using variables that were potentially associated with extent of participation but not included in the primary analyses (e.g., employment status, marital status, overall health status, number of online surveys completed in the last four months, number of online surveys on smoking completed in last month, and an array of reasons for considering quitting that were not directly related to HWLS). Because perceived threat to freedom is often used as a subcomponent of reactance rather than as a manipulation check, additional sensitivity analysis was conducted using a composite measure of reactance combining anger and perceived threat to freedom. In models that included propensity scores or a composite measure of reactance, the results from each adjusted model reported in this paper were consistent in their direction, magnitude, and significance.

## Results

### Sample Characteristics

Table 1 shows the characteristics of the analytic sample for each country. Compared to samples from other countries, the US sample reported the lowest levels of affective state reactance and was least likely to report avoidance. The US participants were also younger, had higher educational attainment, and were more likely to report forgoing cigarettes due to HWLs, compared to Australian and Canadian samples. Compared to the Mexican sample, the US sample was older, had lower educational attainment, and was less likely to report forgoing cigarettes due to HWLs. Within Canada and the US, the level of affective state reactance was no different for the analytic sample compared to the sample that was ineligible due to participation in only one survey wave. For Australia, participants in the analytic sample reported lower affective state reactance than ineligible participants, whereas the opposite was true for Mexico.

**Table 1:** Analytic Sample Characteristics by Country and Between-country Differences, % or Mean (SD).

Variable of Interest	Australia	Canada	Mexico	United States	Total
	n = 1889	n = 1787	n = 1695	n = 2088	n = 7459
Age^a^^,^ ^c^^,^ ^m^					
18–24	4%	6%	15%	9%	8%
25–34	20%	20%	31%	29%	25%
35–44	23%	22%	24%	19%	22%
45–54	25%	25%	16%	20%	22%
55–64	26%	24%	12%	20%	21%
Gender					
Female	48%	49%	43%	46%	47%
Education^a^^,^ ^c^^,^ ^m^					
High school or less	32%	25%	22%	24%	26%
Some college or university	38%	44%	16%	36%	34%
University or more	29%	30%	60%	39%	39%
Income^a^^,^ ^c^^,^ ^m^					
Low	22%	23%	31%	21%	24%
Medium	27%	28%	35%	35%	31%
High	49%	47%	32%	43%	43%
Heaviness of Smoking Intensity^a^^,^ ^m^	2.72 (1.62)	2.30 (1.56)	0.81 (1.23)	2.32 (1.55)	2.08 (1.66)
Recent quit attempt^m^	35%	38%	52%	37%	40%
Quit intentions^m^	41%	42%	46%	40%	42%
Self-efficacy^a^^,^ ^m^	4.85 (2.21)	5.04 (2.10)	5.52 (2.10)	5.01 (2.22)	5.09 (2.18)
Freedom to threat^a^^,^ ^c^	4.42 (1.73)	4.00 (1.79)	3.46 (1.89)	3.44 (1.84)	3.83 (1.86)
Affective state reactance^a^^,^ ^c^^,^ ^m^	3.87 (1.86)	3.62 (1.85)	3.33 (1.96)	3.10 (1.82)	3.47 (1.89)
Attention to HWLs^m^					
Never	32%	28%	10%	37%	27%
Rarely	31%	33%	26%	26%	29%
Sometimes	23%	25%	33%	20%	25%
Often	8%	7%	20%	9%	11%
Very Often	3%	4%	8%	5%	5%
Thinking about health risks^a^^,^ ^m^					
Low	44%	41%	20%	41%	37%
Moderate	31%	31%	27%	29%	30%
High	23%	26%	52%	29%	32%
Avoiding HWLs^a^^,^ ^c^^,^ ^m^	31%	30%	40%	21%	30%
Forgoing cigarettes due to HWLs^a^^,^ ^c^^,^ ^m^	23%	21%	43%	29%	29%

^a^p<0.05 for U.S. vs. Australian sample,

^c^U.S. vs. Canadian sample,

^m^U.S. vs. Mexican sample

### Affective State Reactance and HWL responses

In each country, affective state reactance was strongly correlated with threat to freedom (r = 0.65 in Australia; r = 0.67 in Canada; r = 0.73 in Mexico; r = 0.70 in US), suggesting construct validity of the reactance measure. To test H1 that stronger reactance is associated with more maladaptive responses to HWLs (i.e. lower attention to HWLs, lower cognitive elaboration of risks, more avoiding HWLs, and lower forgoing cigarettes due to HWLs), HWL responses were regressed on study variables, pooling data from all countries. Interactions between reactance and country were statistically significant for all fully adjusted models (i.e., range of p-values for interaction: p = 0.000 to 0.009, with the US as reference group); hence, models were stratified by country (Table 2). In contrary with H1a, stronger affective state reactance was associated with more frequent reading of HWLs and thinking about risks due to HWLs in the US (β_adj_ = 0.08, p<0.001; β = 0.16, p<0.001, respectively), but not in other countries. Smokers with relatively stronger affective state reactance reported less frequent thinking about health risks in Canada as hypothesized in H1b, but this relationship was statistically significant only in adjusted models (β = -0.08, p<0.05). Across all countries, stronger affective state reactance was significantly associated with greater likelihood of avoiding HWLs, supporting H1c. As opposed to H1d, stronger affective state reactance was also significantly associated with forgoing cigarettes due to HWLs. The exception was the lack of association between affective state reactance and forgoing cigarettes in Mexico.

**Table 2:** Bivariate and adjusted associations between state reactance and HWL responses.

	Attention to HWLs		Thinking about health risks due to HWLs		Avoiding HWLs		Forgoing cigarette due to HWLs	
	b	b*	b	b*	OR	AOR*	OR	AOR*
Australia	0.01	0.00	0.01	0.00	1.23^c^	1.26^c^	1.10^c^	1.09^a^
Canada	0.00	-0.02	-0.01	-0.02^a^	1.22^c^	1.18^c^	1.09^b^	1.09^a^
Mexico	-0.02	-0.03	-0.01	-0.01	1.21^c^	1.21^c^	1.04	1.03
United States	0.11^c^	0.08^c^	0.08^c^	0.06^c^	1.47^c^	1.39^c^	1.39^c^	1.37^c^
Pooled	0.02^b^	0.01^a^	0.01^a^	0.00	1.26^c^	1.26^c^	1.12^c^	1.13^c^

^a^ = p<0.05;

^b^ = p<0.01;

^c^ = p<0.001

*Models adjust for country, age, gender, education, income, heaviness of smoking intensity, quit attempt in prior 4 months, quit intentions in next 6 months, self-efficacy, survey wave, and the number of prior surveys completed.

H4 proposed that the relationship between affective state reactance and study outcomes (i.e. HWL responses and smoking cessation) will be weaker in the US with text-only HWLs than other countries with pictorial HWLs. The strongest relationship between affective state reactance and HWL responses was observed in the US as shown in Table 2, partly rejecting H4.

### Subsequent Quit Attempts

Across countries, 45% of the sample attempted to quit smoking over the subsequent follow-up period. Inconsistent with H2 that stronger affective state reactance will be associated with less quit attempts at follow-up, greater affective state reactance was significantly associated with greater likelihood of making a quit attempt in the bivariate model (OR = 1.04, p<0.01), as well as in the two adjusted models that included attention to HWLs and cognitive elaboration due to HWLs (Table 3). Higher attention to HWLs, cognitive elaboration due to HWLs, and exhibiting behavioral responses to HWLs, were independent predictors of greater likelihood of attempting to quit. Model results suggested that higher self-efficacy, being older, having recently made a quit attempt, and intentions to quit were associated with greater likelihood of subsequent quit attempts. H3 and H5 stated that affective state reactance and self-efficacy will moderate the effects of HWLs responses on downstream cessation. In H4, we hypothesized that the relationship between affective state reactance and smoking cessation will be stronger in countries with pictorial HWLs than in the US which have text-only HWLs. All the hypotheses were rejected in the adjusted models, where interactions between affective state reactance and other study variables (i.e., country, self-efficacy, attention to HWLs, cognitive elaboration due to HWLs, behavioral responses to HWLs) were not statistically significant.

**Table 3:** Predictors of subsequent quit attempts.

Independent Variables	%	Bivariate	Adjusted 1	Adjusted 2	Adjusted 3	Adjusted 4
		OR	AOR	AOR	AOR	AOR
Country						
United States	42%	REF	REF	REF	REF	REF
Australia	40%	0.92	1.07	1.08	1.03	1.14
Canada	42%	0.97	0.99	0.96	0.96	1.10
Mexico	59%	1.83^c^	1.16	1.18	1.21^a^	1.31^b^
Age						
18–24	57%	REF	REF	REF	REF	REF
25–34	56%	0.96	0.95	0.94	0.95	0.96
35–44	48%	0.68^c^	0.84	0.78	0.80	0.87
45–54	38%	0.47^c^	0.74^a^	0.69^b^	0.70^b^	0.79
55–64	34%	0.38^c^	0.63^b^	0.59^c^	0.57^c^	0.69^b^
Gender						
Male	46%	REF	REF	REF	REF	REF
Female	45%	0.87^a^	1.00	0.98	0.96	1.01
Education						
High school or less	39%	REF	REF	REF	REF	REF
Some college or university	40%	1.06	1.06	1.07	1.05	1.06
University or more	54%	1.72^c^	1.27^b^	1.27^b^	1.26^b^	1.17
Income						
Low	44%	REF	REF	REF	REF	REF
Medium	45%	1.11	0.98	0.99	0.98	1.01
High	47%	1.17^a^	0.94	0.96	0.95	0.97
Heaviness of Smoking Intensity	1.68^	0.80^c^	0.95^a^	0.96	0.95	0.96
Recent Quit attempt						
No	21%	REF	REF	REF	REF	REF
Yes	76%	13.39^c^	6.89^c^	6.62^c^	6.83^c^	6.60^c^
Quit Intentions						
No	26%	REF	REF	REF	REF	REF
Yes	67%	3.53^c^	2.21^c^	2.10^c^	2.15^c^	2.16^c^
Self-efficacy	5.59^	1.15^c^	1.04^b^	1.04^b^	1.06^c^	1.03^a^
State reactance	3.59^	1.04^b^	1.03^a^	1.03^a^	1.01	1.01
Attention to HWLs						
Never	33%	REF	REF	N/A**	N/A***	N/A****
Rarely	39%	1.17^b^	1.24^b^			
Sometimes	53%	1.69^c^	1.55^c^			
Often	64%	2.26^c^	1.98^c^			
Very Often	69%	2.58^c^	2.39^c^			
Thinking about health risks due to HWLs						
Low	27%	REF	N/A*	REF	N/A***	N/A****
Moderate	42%	1.64^c^		1.30^e^		
High	63%	3.07^c^		1.83^f^		
Avoiding HWLs						
No	36%	REF	N/A*	N/A**	REF	N/A****
Yes	59%	1.90^c^			1.63^c^	
Forgoing cigarettes due to HWLs						
Never	32%	REF	N/A*	N/A**	N/A***	REF
Once or more	70%	3.26^c^				2.37^c^

^a^ = p<0.05;

^b^ = p<0.01;

^c^ = p<0.001

^ = mean

*Model adjusts for all variables listed in the table, survey wave, and time in sample, but not avoiding HWLs, thinking about health risks due to HWLs, and forgoing cigarette due to HWLs

**Model adjusts for all variables listed in the table, survey wave, and time in sample, but not attention to HWLs, thinking about health risks due to HWLs, and forgoing cigarette due to HWLs

***Model adjusts for all variables listed in the table, survey wave, and time in sample, but not attention to HWLs, avoiding HWLs, and forgoing cigarette due to HWLs

****Model adjusts for all variables listed in the table, survey wave, and time in sample, but not attention to HWLs, thinking about health risks due to HWLs, and avoiding HWLs

## Discussion

Our analysis found little evidence to support H1a that smokers who report higher levels of affective state reactance in response to cigarette HWLs are less likely to attend to or think about health risks due to HWLs compared to smokers who report lower levels of reactance. In countries with pictorial HWLs, reactance was not associated with either of these HWL responses. The exception was for Canada, where reactance had a marginally inverse association with elaboration of risks, although only when adjusted for potential adjustment variables. Canada’s HWL policy differs from other countries because it combines pictorial HWLs on packs that illustrate smoking-related risks along with package inserts (i.e., small leaflets inside of packs) that include efficacy enhancing messages that appear to promote cessation behavior [37, 40]. These kinds of efficacy messages aim to minimize message rejection and potentiate behavior change that is based on fear appeals. However, our study protocol did not involve showing participants these inserts before querying affective state reactance. Showing participants the HWLs in combination with inserts, as happens under natural exposure conditions, may not have produced the same effects. However, the negative association between reactance and elaboration was marginal, was not observed in other countries, and was not found for other HWL outcomes. In the context of repeated exposure to HWLs, reactance may be one component of negative affect, which some experiments have found to be the mediational pathway towards increased perceived risk [26] and intentions to quit [4, 41]. Nevertheless, future research may benefit from exploring the joint impact of HWLs and package inserts or other similar messaging that aims to enhance efficacy.

In all countries, stronger reactance to HWLs was associated with greater likelihood of forgoing cigarettes due to HWLs, which was the opposite of what was predicted in H1b. The associations observed between reactance and avoiding HWLs in all countries as hypothesized in H1c are, at first glance, suggestive of counterproductive, defensive responding. However, avoiding HWLs was associated with greater likelihood of subsequent quit attempts across countries, which is consistent with another observational study [42]. Two additional observational studies with longer intervals between survey waves have found that smokers who report avoiding HWLs are equally likely to quit as other smokers who do not avoid HWLs [27, 30]. Overall, these studies are consistent with research on “ironic processes” that can accompany attempts to suppress thoughts. Studies across a range of contexts find that attempts to suppress thoughts often make the thoughts more likely to arise in the person attempting to suppress them [43]. Hence, experimental research that has found that smokers often direct their attention away from pictorial HWLs [24] is not necessarily indicative of diminished or counterproductive effects.

Data from the current study indicated that the average level of reactance toward HWLs appeared lower in the US than in the other countries, which is unsurprising given the lower threat posed by the relatively old, textual content of HWLs compared to the more novel text and pictorial imagery used on HWLs in Australia, Canada, and Mexico. However, reactance elicited amongst US participants through the process of rating their HWLs was more strongly associated with attention to and elaboration of risks due to HWLs than amongst participants from other countries. This is consistent with previous research showing that, although relatively fewer US smokers attend to HWLs, this can nevertheless lead to smoking cessation [28]. The stronger effects for reactance in the US could be explained by a number of factors. First, smokers who report relatively strong reactance to weaker, older, text-only HWLs may also have a stronger personal tendency to engage with warning label messages, including experience of negative affect actually promotes health-protective behavior. Similarly, the reactance elicited from text-only HWLs may imply more processing effort than the reactance aroused by prominent pictorial HWLs, thereby strengthening the association between reactance and adaptive responses.

Our results indicate that stronger reactance to HWLs did not hamper subsequent quitting attempts, contrary to H2; in fact, reactance was positively associated with cessation attempts in some models. Reactance was no longer an independent predictor of cessation attempts only in adjusted models that included behavioral responses to HWLs (i.e., avoidance, foregoing cigarettes due to HWLs). This suggests that these behaviors may mediate the effects of reactance. Contrary to H3 or H5, we did not find any statistically significant interactions between reactance and four psychological and behavioral responses to HWLs or self-efficacy to quit, suggesting that the influence of reactance on future quit attempts does not differ by the strength of responses to HWLs or self-efficacy. In other words, reactance appears not to have any counterproductive effects on quitting behavior, even amongst smokers who have weak responses to HWLs or whose self-efficacy to quit is low. These results are similar to prior experimental and observational studies that have assessed the role of trait reactance on the effect of HWL in increasing quit intention and quit attempts [32, 33]. Moreover, we found no significant interaction between reactance and country, rejecting H5 and concluding that the effect of affective state reactance on subsequent quit attempts does not differ by country. In the end, these results suggest that future communication research must go beyond examining reactance as a primary dependent variable. As was found in the current study, the maladaptive effects of affective state reactance cannot be assumed, and therefore warrants more nuanced inquiry. For example, future studies may benefit by using longitudinal methods to examine the intervening role of reactance in explaining the relationship between explicit HWL content manipulations and downstream outcomes such as behaviors.

Our findings are generally consistent with Context, Executive and Operational Systems (CEOS) theory [29] and research on the affect heuristic [25, 44, 45] which argue that affect, whether conscious or unconscious, motivates behavior when it is sufficiently strong, particularly when affect also supports competing behaviors (e.g. the positive experiences attained from smoking, positive marketing messages). These models suggest that strong negative affect associated with smoking, for which affective state reactance may be one of a variety of indicators, can lead to increased quitting behavior. On any particular occasion, the options with regard to HWLs are to avoid HWLs, to react against them, or to take action to remove the source of the negative affect (i.e. to make a quit attempt). According to CEOS theory, however, it is unlikely that anyone would always react against HWLs, and the occasions they do not are the base conditions to stimulate action. For instance, some experimental studies that briefly expose smokers to graphic HWLs have found that HWLs may work best for smokers with high self-efficacy [46]. On the contrary, in population studies where smokers are repeatedly exposed to HWLs, smokers may attend more to HWLs when they feel capable of quitting, with levels of self-efficacy and motivation to quit fluctuating over time. Thus, while some experimental studies which explore specific reactions to isolated events may find inverse associations between negative affect and proxies for smoking cessation, population studies which involve accumulated exposures to HWLs over a large number of events show a different response.

Our study has several limitations. First, the causal inference between reactance and four psychological and behavioral HWL responses may be limited due to the cross-sectional analysis of the association. Although we assumed that reactance toward specific HWLs is likely to be similar to that which occurs when encountering HWLs in daily life and therefore preceded four psychological and behavioral HWL responses, future studies may want to examine temporal relationships using a longitudinal design. Second, because of limited resources, the measurement of state reactance did not include assessment of negative cognitions using the thought-listing technique, which is traditionally measured along with the approach we used [47]. Nonetheless, prior research indicates the affective, anger component of reactance that we measured captures the bulk of the reactance construct [22]. Also, the high correlation we found between affective state reactance and freedom threat (range of r = 0.64 to 0.73) supports the construct validity of the reactance scale. Furthermore, our results are consistent with prior studies of trait reactance and HWL responses [32, 33], similar to the consistencies between trait and state reactance in other communication areas [21]. Hence, inclusion of the cognitive component of reactance is unlikely to have substantially changed our results, including the reversal of the positive effects that we report here. However, future research should nevertheless consider the cognitive component of reactance, as well as enriched measurement of affective responses to HWLs. Enriched measurement in these domains will be necessary to test hypotheses that reactance works similarly to other negative affect, by promoting adaptive responses to HWLs. Future measurement approaches should also consider measurement of reactance in more naturalistic conditions of exposure, as we assessed reactance immediately after forced exposure to pictorial HWLs on packaging at the time of the survey. More naturalistic data collection techniques, such as ecological momentary assessment, may prove necessary to better understand reactance under conditions of repeated exposure to HWLs.

Differences between the analytic sample and the sample that was not followed up suggest that attrition bias may limit the generalizability of our results. However, the sample that was not followed generally reported stronger four psychological and behavioral HWL responses, and therefore we may have underestimated HWL effects. Nevertheless, we obtained very similar results when we adjusted models for propensity scores that accounted for a variety of factors associated with attrition. Hence, attrition bias does not appear to have strongly influenced our results or their interpretation. The generalizability of our study is also potentially limited due to its unknown sampling frame, with panel participants assembled in ways that may have varied systematically across countries. High internet penetration rates in 2013 in Australia (83%), Canada (85%), and the U.S. (84%) somewhat limit concerns about differential participation by internet accessibility [48]. The relatively low internet penetration rate in Mexico (43%), however, likely led to the Mexican sample over-representing smokers from higher socioeconomic status groups, even though they were purposefully selected to represent key consumer groups [48]. Because of differences in sample composition across countries, comparisons across countries should be interpreted cautiously. Future studies may thus be needed to verify our study results in population-based representative samples. Finally, differences in HWL characteristics across countries may account for some of the effects that we found. In spite of this, however, the generally consistent pattern of results across countries with different HWL content suggests that similar processes operate independent of HWL content.

## Conclusions

Overall, this study suggests that HWLs are effective in increasing smoking cessation, even for smokers with high affective state reactance. Based on the evidence, policy makers should not be reluctant to implement prominent HWLs because of their seemingly short-term negative psychological or behavioral impacts, given their long-term positive impact on smoking cessation.
